# Surface Wettability of ZnO-Loaded TiO_2_ Nanotube Array Layers

**DOI:** 10.3390/nano10101901

**Published:** 2020-09-23

**Authors:** Marius Dobromir, Claudia Teodora Konrad-Soare, George Stoian, Alina Semchenko, Dmitry Kovalenko, Dumitru Luca

**Affiliations:** 1 Department of Research, Faculty of Physics, Alexandru Ioan Cuza University of Iaşi, 11, Carol I Blvd., 700506 Iaşi, Romania; 2 Faculty of Physics, Alexandru Ioan Cuza University of Iaşi, 11, Carol I Blvd., 700506 Iaşi, Romania; claudia.konradsoare@gmail.com; 3 National Institute of Research and Development for Technical Physics, 47, Dimitrie Mangeron Blvd., 700050 Iaşi, Romania; gstoian@phys-iasi.ro; 4 Faculty of Physics and Information Technology, Francisk Skorina Gomel State University, Sovetskaya Str. 104, 246019 Gomel, Belarus; alina@gsu.by (A.S.); dkov@gsu.by (D.K.)

**Keywords:** TiO_2_ nanotube array layers, ZnO loading, reactive RF magnetron sputtering, ZnO/TiO_2_ heterojunction, surface wettability, back reaction

## Abstract

Herein we report on the synthesis and the effects of gradual loading of TiO_2_ nanotube array layers with ZnO upon surface wettability. Two-step preparation was chosen, where TiO_2_ nanotube layers, grown in a first instance by anodization of a Ti foil, were gradually loaded with controlled amounts of ZnO using the reactive RF magnetron sputtering. After crystallization annealing, the formerly amorphous TiO_2_ nanotubes were converted to predominantly anatase crystalline phase, as detected by XRD measurements. The as-prepared nanotubes exhibited a well-aligned columnar structure, 1.6 μm long and 88 nm in diameter, and a small concentration of oxygen vacancies. Ti^2+^ and Ti^3+^ occur along with the Ti^4+^ state upon sputter-cleaning the layer surfaces from contaminants. The Ti^2+^ and Ti^3+^ signals diminish with gradual ZnO loading. As demonstrated by the VB-XPS data, the ZnO loading is accompanied by a slight narrowing of the band gap of the materials. A combined effect of material modification and surface roughness was taken into consideration to explain the evolution of surface super-hydrophilicity of the materials under UV irradiation. The loading process resulted in increasing surface wettability with approx. 33%, and in a drastic extension of activation decay, which clearly points out to the effect of ZnO-TiO_2_ heterojunctions.

## 1. Introduction

Titanium dioxide (TiO_2_) is a non-toxic, chemically stable, highly active photocatalytic oxide semiconductor. It can be photo-activated by UV light with energy in excess of the band gap (3.0–3.2 eV) and used for pollutant degradation in low-cost, environment-friendly applications.

New opportunities for application of TiO_2_ materials occurred in different areas, such as dye-sensitized solar cells (DSSCs) and electrochemical cells, catalysis, supercapacitors [1,2,3,4,5], gas sensors [6], biomaterials [7], and environmental and energy applications [1,8]. Large specific area is a key factor for improving the degradation rate and catalytic efficiency at the catalyst/organic pollutant interface. This requirement is fulfilled by both nanopowders and bi-dimensional nanostructures. Nanotubes feature particularly large area/volume ratio and faster electron transport, as well as low recombination rate of charge carriers. This enables increased photocatalytic efficiency and durability [9,10,11,12,13,14,15,16].

TiO_2_ materials feature low electrical conductivity and high recombination rates of charge carriers. To overcome these drawbacks, doping of these materials with small amounts of anion or cation species, as well as the formation of nano-heterojunctions with other appropriate oxide materials, were proposed as the main approaches [17,18]. Loading of the TiO_2_ nanotube surface with ultrathin oxide layers or nanoparticles may result in the formation of semiconductor heterojunctions, which enhances the spatial separation of electron-hole pairs, thus reducing the recombination rate [17,19,20,21,22]. Several methods for ZnO/TiO_2_ heterojunctions fabrication were reported, such as: (a) formation of ZnO-TiO_2_ nanocomposite arrays on Ti fabric using hydrothermal process [20]; (b) decorating of TiO_2_ nanotube surface with nano-rice-shaped ZnO using chemical bath deposition [23]; (c) growth of ZnO nanostructures on top of TiO_2_ nanotubes via electrochemical method [24,25] or dip coating [26]; and (d) deposition of heterojunction of ZnO on hydrogenated TiO_2_ nanotube arrays by atomic layer deposition (ALD) [27]. The association of ZnO to alter the band structure of the TiO_2_ and surface hydrophilicity is of particular interest, due to close band gap energy values of ZnO and TiO_2_, and, in the case of ZnO, higher electron mobility for fast charge transport and lower carrier pair lifetime [28].

The preparation of ZnO/TiO_2_ heterojunctions using the RF sputtering as material sources to load the nanotube surface with ultra-thin ZnO layers or individual nanoparticles is scarcely reported. Nevertheless, this latter technique has been documented as an easy means to control materials morphology, structure, elemental and chemical composition, and wetting behaviour by facile adjustment of the sputtering and deposition parameters.This results in better film adhesion, uniformity, high deposition rate, and efficiency [29,30,31]. Recently, Yan et al. [32] used the magnetron sputtering to grow ZnO/TiO_2_ heterostructures by sputter-depositing *TiO_2_* nanowires on top of ZnO nanorods.

We report here on the characterization of the ZnO/TiO_2_ heterojunctions fabricated by sputtering the ZnO on top of anodized TiO_2_ nanotube array layers. Experiments were done to assess surface morphology, crystallinity, and elemental and chemical composition. The results are discussed in correlation with the surface wetting characteristics, tacking into account the synergistic effects of surface chemistry, texture and increased spatial separation of the electro-hole pairs inside the synthesized ZnO/TiO_2_ heterojunctions. The peculiar presence of Ti_2_O_3_ and TiO suboxides, which coexist with the main TiO_2_ component in the surface region of sputter-cleaned nanotube surface, is also discussed, in correlation with the gradual loading of the nanotubes.

## 2. Materials and Methods

TiO_2_ nanotube arrays layers were prepared by electrochemical anodization of a 0.25-mm-thick Ti foils (Aldrich, 99.7% purity). Nanotube array samples were prepared in an electrochemical bath containing 0.15M NH_4_F in ethylene glycol, under 30 V biasing voltage and magnetic stirring (400 rpm) conditions for 3 h. The ZnO thin films were deposited on top of the TiO_2_ nanotube arrays, at room temperature, in an RF (13.56 MHz) magnetron sputtering deposition facility (base pressure of 2×10^−3^ Pa). The reactive magnetron discharge was conducted in Ar + O_2_ gas mixture with a 7.62 cm diameter Zn (Aldrich, 99.995% purity) disk cathode and grounded sample holder. During deposition, the total pressure in the discharge was kept at 2.7 Pa, by introducing 5 sccm Ar and 1 sccm O_2_ gas, via automatic mass flow rate (MFR) controllers.

Variable thickness ZnO films were deposited on top of the TiO_2_ nanotube arrays for deposition durations of 30, 60 and 90 min. Therefore, the samples were labeled as: TZN_0 (the reference sample), TZN_30, TZN_60, and TZN_90, respectively. The surface morphology was studied by field emission scanning electron microscopy (FESEM) using a JEOL JSM 6390 equipment (JEOL, Oberkochen, Germany) and by atomic force microscopy (AFM) using a NT-MDT Solver Pro M instrument (NT-MDT Co., Zelenograd, Russia), operated in tapping mode. The data from the AFM images were interpreted by using the Gwyddion software (ver. 2.41).

The anodized nanotube layers were amorphous, as confirmed by the X-ray diffraction (XRD) data collected with a Shimadzu LabX XRD-6000 diffractometer (Shimadzu, Columbia, MD, USA) (CuKα radiation, λ = 1.54182 Å), operated in a Bragg-Brentano arrangement. Thereafter, the crystallization annealing of the nanotubes was done at 450 °C, for 1 h, in air, in a Barnstead Thermolyne 1300 furnace.

Information on surface elemental composition and chemical states of the elements present at the sample surface was derived from the XPS measurements carried out using a PHI 5000 VersaProbe photoelectron spectrometer (Ulvac-PHI, Inc., Chikasaki, Japan). The XPS spectra were recorded using monochromated Al K*α* radiation (1486.7 eV), and the C *1s* core level signal (*BE* = 284.6 eV) was used for calibration of the binding energies in the XPS spectra. Peak deconvolution of the high-resolution XPS spectra has been done using the PHI MultiPak software (ver. 9.6, Ulvac-PHI, Inc., Chikasaki, Japan) in order to identify the oxidation states and the types of atomic binding. The binding energy values were accurate within ± 0.2 eV.

Static contact angle measurements (CA) were conducted for surface wettability quantification during both surface activation and back-reaction. CA measurements have been done by means of a Data Physics OCA 15EC goniometer (Filderstadt, Germany), at room temperature and 50% relative humidity, in a sessile drop arrangement. The average values of CA were calculated from five different CA measurements at different locations on the sample’s surface. Water drop volumes of 0.5 μL were used to avoid gravity-induced drop shape alteration and to diminish the effects of evaporation effects during CA measurements. For photoactivation, the layers’ surfaces were irradiated with optical radiation from a non-filtered high-pressure Hg lamp (UV irradiation 120 mW/cm^2^, λpeak = 253 nm) with emission in both UV and visible range, as described elsewhere [33].

## 3. Results and Discussion

### 3.1. Surface Morphology. SEM and AFM Results

**Figure 1:**
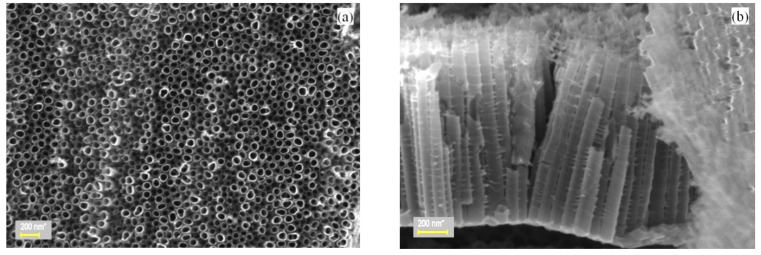

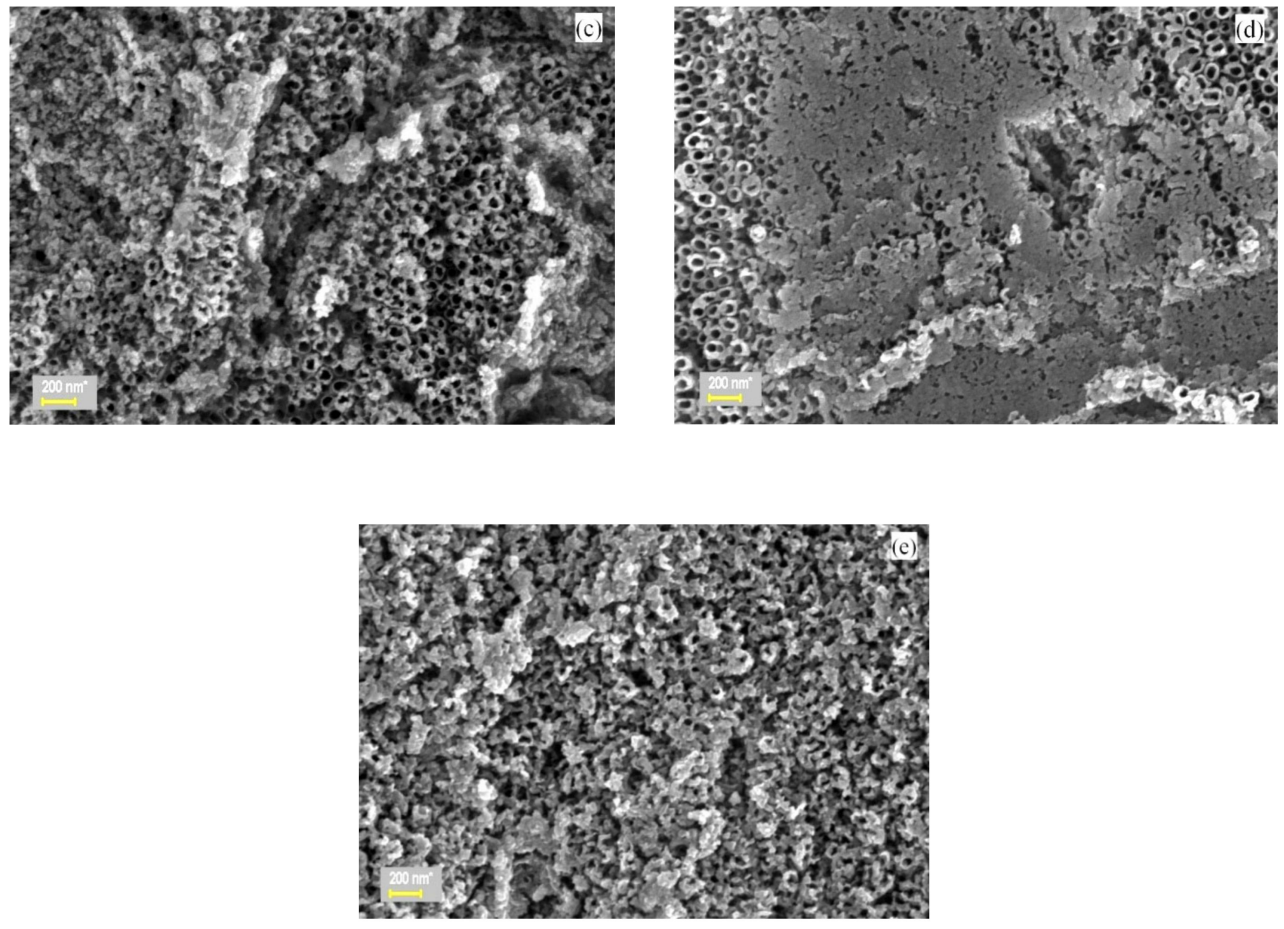
SEM images of: (**a**) the top and (**b**) cross-section surfaces of the TZN_0 sample. Top view of samples (**c)** TZN_30, (**d**) TZN_60, and (**e**) TZN_90. The overall magnification is 24,100 ×.

The SEM micrographs of the investigated samples are shown in Figure 1. Well-aligned nanotube structures 1.6 μm long and 88 nm in diameter, normal to the Ti foil underlayer surface, are visible in the images of the bare TiO_2_ nanotube array layer (Figure 1a,b).

A gradual loading of the TiO_2_ nanotube surface with ZnO is evident, starting from the SEM images presented throughout Figure 1c–e, which ends in nearly full ZnO coverage after sputter-deposition time of 90 min. Microscopical cracks occur occasionally in the cross-section SEM images of the TiO_2_ nanotubes, as depicted in Figure 1c. They accommodate large amounts of ZnO particles with similar shapes as on top and inside the nanotube arrays [27]. Such cracks occur at grain boundaries of the Ti foil after electrochemical anodization.

**Figure 2:**
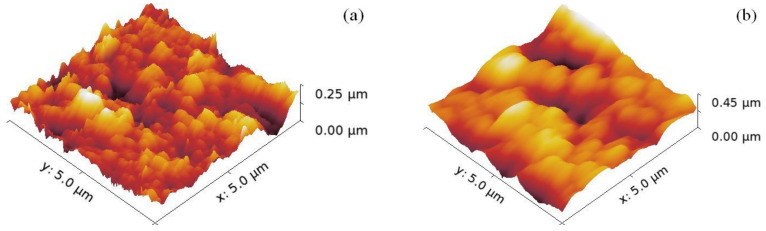

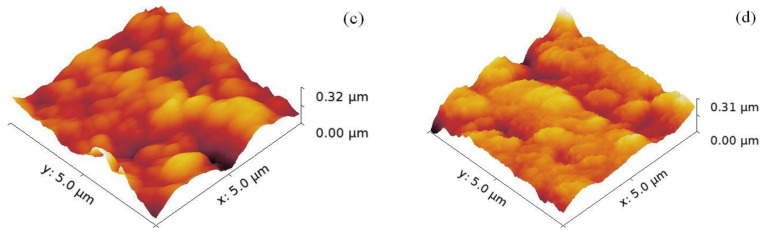
3D AFM images of: (**a**) the reference sample, (**b**) TZN_30, (**c**) TZN_60, and (**d**) TZN_90.

The root mean square roughness (RMS) values were derived from the AFM images of the loaded surfaces, scanned over a (5 × 5) μm^2^ area. Such typical AFM images are depicted in Figure 2, where no macroscopical cracks were imaged.

As shown in Table 1, except for the sample TZN_30 (where RMS = 63.20 nm), the RMS values of the other samples fluctuate in a narrow range (34.4–37.4 nm).

### 3.2. Layer Crystallinity. XRD Results

**Figure 3:**
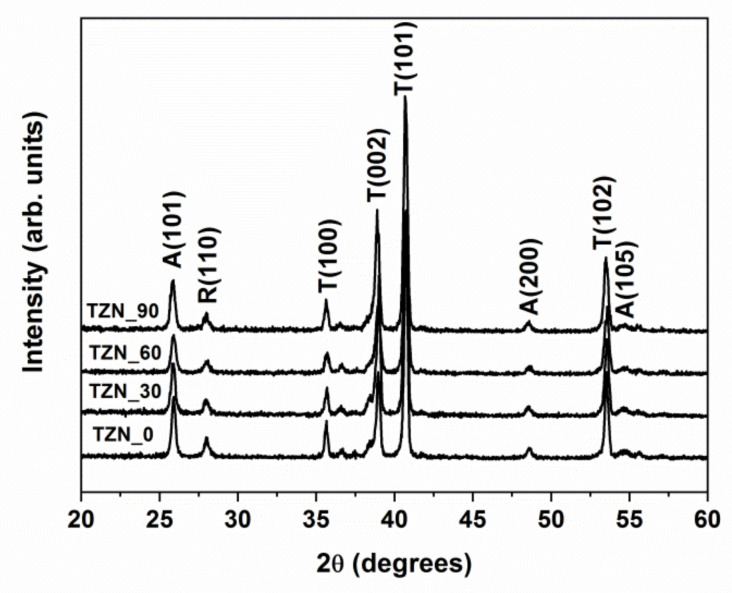
X-ray diffraction patterns of the investigated samples.

The X-ray diffraction patterns of the prepared samples are depicted in Figure 3, where three distinct peaks are evident, at 2*θ* = 25.30°, 48.04° and 53.88°, corresponding to the reflections on the A(101), A(200) and A(105) crystalline planes of the anatase phase. A small diffraction peak, assigned to the reflection on the TiO_2_ rutile R(110) planes, occurs at 2*θ* = 27.43°. The diffraction peaks at 2*θ* = 35.05°, 38.42°, 40.16°, and 52.98° originate in the reflections on the Ti planes, namely T(100), T(002), T(101), and T(102), respectively.

The XRD patterns of the ZnO-loaded TiO_2_ nanotube array layers show no diffraction peaks of ZnO, due to the small amount of material, as depicted from SEM measurements. However, the presence of the amorphous ZnO phase cannot be ruled out a priori. The patterns of the sputtered ZnO thin films grown at room temperature show the characteristic peaks of hexagonal wurtzite structure, with strong (002) reflection, which indicates preferential growth orientation perpendicular to the substrate [33,34,35,36].

The weight percentage of the anatase phase, W_A_, was determined using the Spurr–Myers formula [37]:

(1) $$W_{A}\;=\;I_{A}/\left(I_{A}+1.256I_{R}\right)$$

where *I_A_* and *I_R_* are the intensities of the anatase A(101) and rutile R(110) XRD peaks. As seen in Table 1, this quantity fluctuates between 70.2 and 72.6%.

The TiO_2_ grain size was calculated from the XRD patterns, according to the Debye–Scherrer formula [38]:

(2) $$D\;=\;\frac{0.9\lambda }{\beta cos\theta }$$

where *β* is the diffraction half-height peak width at the Bragg angle *θ,* and λ is the wavelength of the X-ray radiation. The average crystalline grain size in the TiO_2_ nanotube wall, derived from the A(101) signal using Equation (2), fluctuates between 26.1 nm and 22.6 nm (see Table 1), i.e. comparable or smaller than the thickness of the nanotube wall. This was confirmed by previous reports, where annealing at 500 °C results in the increase of the grain size up to 200 nm, i.e., comparable with the full nanotube length [39].

### 3.3. Elemental and Chemical Composition. XPS Results

**Figure 4:**
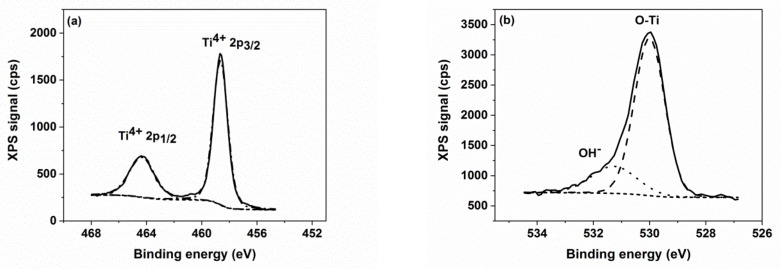

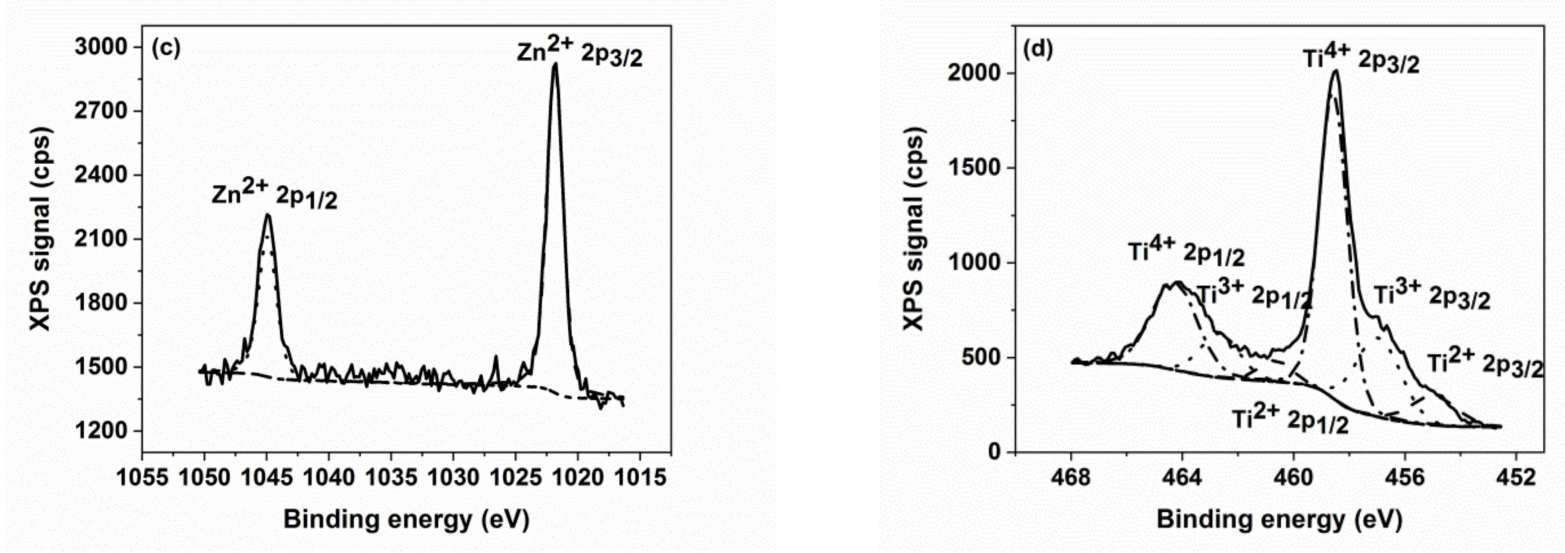
High-resolution XPS spectra of: (**a**) Ti *2p* and (**b**) O *1s* of the as-prepared layers; the spectra of *Zn 2p and Ti 2p* of the sputtered samples are depicted in panes (**c**) and (**d**), respectively.

As a general rule, the XPS spectra of the investigated samples show slight differences in the high-resolution area peaks. Therefore, we will further discuss only the case of the XPS data of the sample TZN_30 (Figure 4). The XPS high-resolution spectra of the as-prepared layers show the signatures of Ti 2*p,* O 1*s* and a significant signal due to C 1*s* (BE = 284.6 eV) contaminant typically originating from the ethylene-glycol-ammonium fluoride anodization electrolyte [40]. The Ti^4+^ signal with Ti 2*p*_3/2_ and Ti 2p_1/2_ peaks (*BE* = 458.8 eV and 464.3 eV, respectively), with a separation of about 5.54 eV [41], is present exclusively in the XPS spectra of the as-prepared nanotubes (Figure 4a), without other reduced states. Additionally, a second contaminant-like layer is formed at the TiO_2_ surface, due to O-H bonds (*BE* = 531.4 eV) [41,42,43], as depicted in Figure 4b.

To remove the contaminants from the surfaces of the samples, a sputter-cleaning Ar^+^ ion bombardment (2 keV, 1 μA) was done in UHV conditions (*p* = 3 × 10^−6^ Pa), followed by subsequent XPS measurements of Zn 2*p* (Figure 4c) and Ti 2*p* (Figure 4d) signals in the final state. While no new relevant changes are present in the Zn 2*p* XPS spectrum of the sputtered samples, the shape of the Ti 2*p* spectrum is significantly changed after Ar^+^ ion bombardment of the Ti surface [42], which acts as a reducing factor, as reported by Hashimoto et al. [44]. The peaks at 456.7 eV and 462.2 eV in Figure 4d are linked to the presence of the Ti *2p_3/2_* and Ti *2p_1/2_* XPS signals of Ti^3+^ state [45]. Additional peaks are present in Figure 4d, at *BE* = 455.1 eV and 460.6 eV, corresponding to Ti *2p_3/2_* and Ti *2p_1/2_* XPS signals ascribed to Ti^2+^ [41].The elemental composition data of the sputtered samples are shown in Table 2.

Thus, apart from the main TiO_2_ component, Ti_2_O_3_ and TiO sub-oxides coexist in the sputtered nanotubes. Their XPS signature decreases with gradual ZnO loading (the Ti^2+^/Ti^4+^ ratio decreases from 0.17 to 0.03 and the Ti^3+^/Ti^4+^ ratio from 0.40 to 0.26, as seen in Table 3). This decrease of the concentration of Ti suboxides can be ascribed to the increasing effect of screening of the sputtering process upon increasing the ZnO coverage.

Apart from the elemental and chemical information on core-level photoelectron lines, the XPS technique allows additional investigation of band gap and valence band localization and structure, despite not being as complete as the ultraviolet photoelectron spectroscopy (UPS) [46,47].

**Figure 5:**
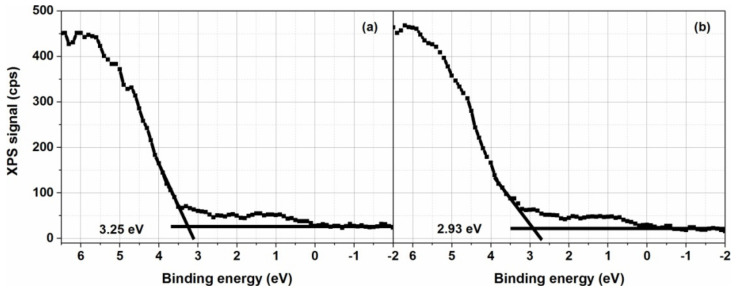
VB-XPS spectra of: (**a**) reference sample, (**b**) TZN_30 sample.

Preliminary characterization of the ZnO/TiO_2_ heterostructures was done here by deriving the position of the valence band maxima (Figure 5). The valence band maximum (VBM) of the bare TiO_2_ layer is located at 3.25 eV below the Fermi level, in agreement with the literature [47], while the VBM of the heterostructure is located at 2.3 eV below the Fermi level, irrespective of the ZnO coverage. Therefore, only one VB-XPS spectrum for the loaded samples was introduced (Figure 5b). The shift in *BE* values is related to the presence of ZnO on top of the TiO_2_ surface, which leads to band gap narrowing [48].

**Figure 6:**
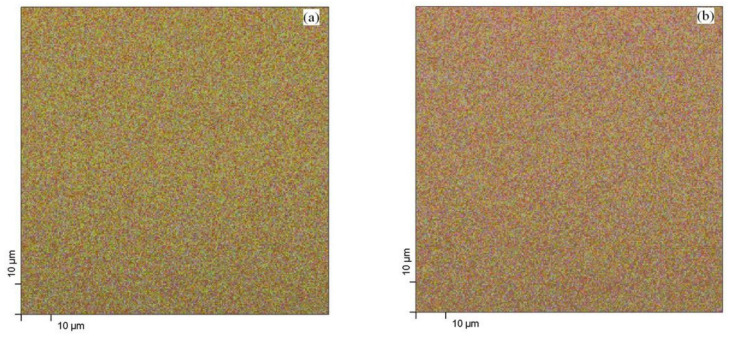

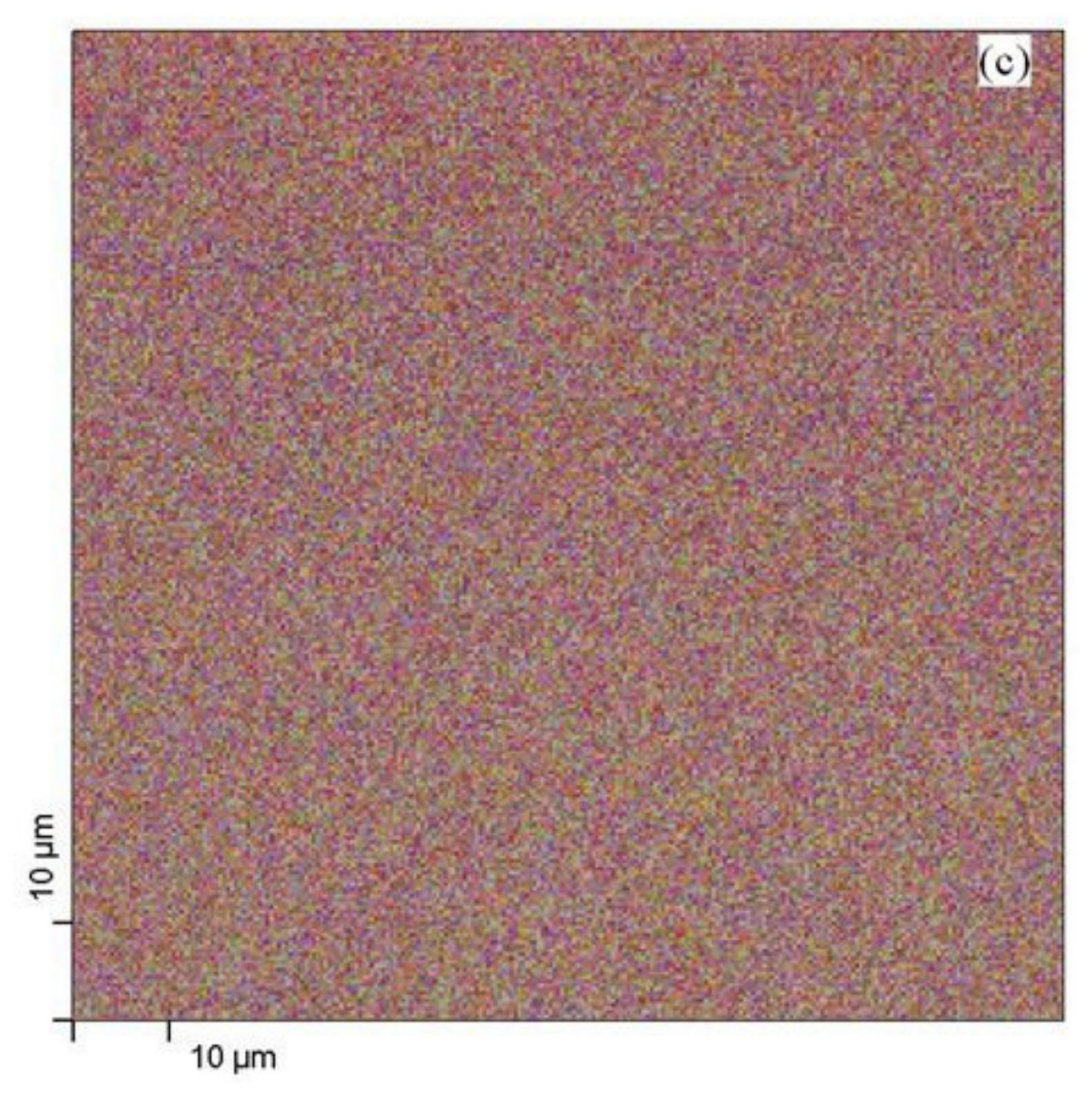
The RGB overlay of O, Ti and Zn distribution in samples: (**a**) TZN_30, (**b**) TZN_60 and (**c**) TZN_90.

Figure 6 shows the surface distribution of the atomic species on the layer surface, as obtained from the XPS chemical mapping. The elemental composition data were collected from an area of (100 × 100) μm^2^ for 256 points/line-scan. The overlayed colors (oxygen in red, titanium in green and zinc in blue) of all the investigated layers are, respectively: (a) TZN_30, (b) TZN_60 and (c) TZN_90 samples.

**Figure 7:**
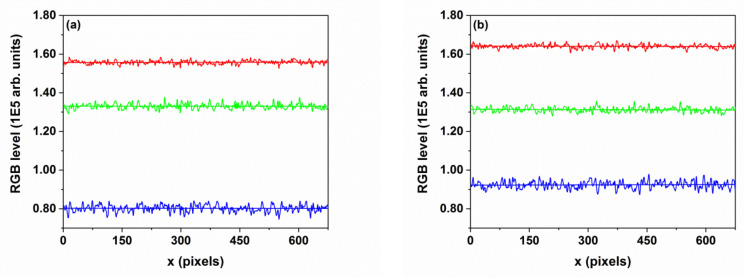

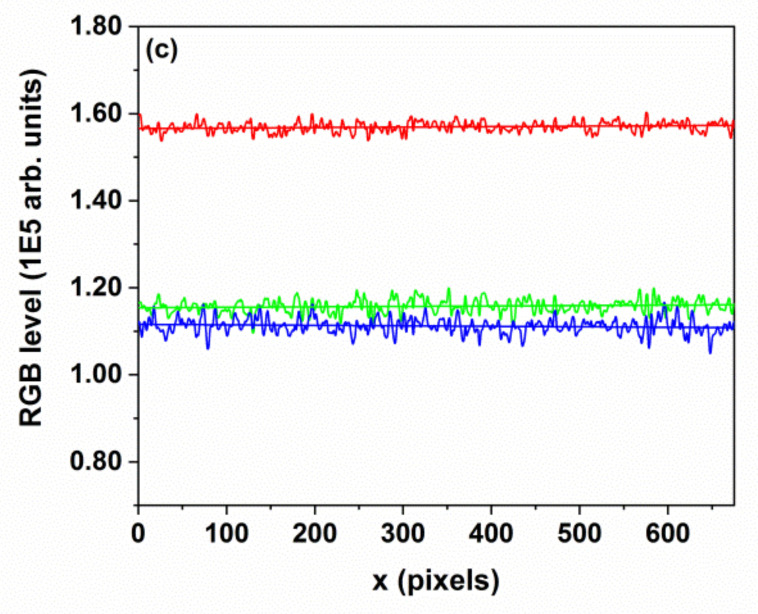
The color profiles extracted from the XPS chemical mapping images for: (**a**) TZN_30, (**b**) TZN_60, and (**c**) TZN_90 samples.

Uniform distribution of the atomic species is shown on a macroscopic scale in all the images in Figure 6, which is sustained by the results shown in Figure 7, where RGB scans were plotted across the chemical maps, with the color coding mentioned previously. The digital images of the XPS chemical mapping were processed by using the *Color Profiler* plugin of the *ImageJ* package. Nearly constant values of the oxygen signal is present in all the samples, while the zinc signal increases gradually with the deposition time (and, consequently, the ZnO coverage) at the expense of the titanium signal (green color). This evolution is due to the specific probing depth of the XPS analysis (5–10 nm).

The results in Figure 7 agree with the atomic concentration values of O, Ti and Zn within the probing depth of the XPS technique (Table 2) and indicate good uniformity of elemental composition over the (100 × 100) μm^2^ macroscopic area.

### 3.4. Surface Wettability. Contact Angle

The wettability of a surface is influenced by its morphology, i.e., by surface roughness and the material-dependent surface energy. For the hydrophilic surfaces, the CA increases with surface roughness [49]. Therefore, apart from changing the surface chemistry, a surface can undergo wettability conversion by modifying its micro and nanostructure [31,49]. Due to the heterogeneous nature of the investigated surfaces, and the changes in surface morphology, the combined effects should be evaluated. Under the mentioned conditions, the photon energy in the incident UV light (4.9 eV) is larger than the band gap of both materials.

**Figure 8:**
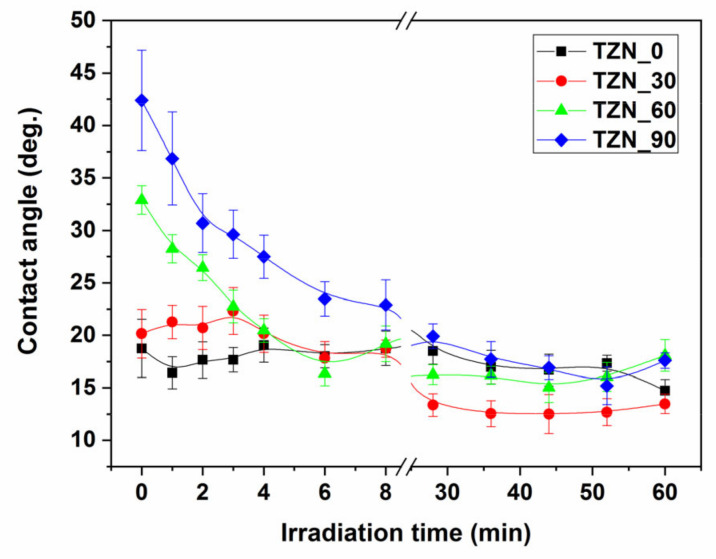
Contact angle vs. irradiation time during surface photo-activation.

The effects of surface microstructure on the wettability can be evaluated by a thorough analysis of the CA values, prior to UV exposure. An inspection of the initial CA values of water with the layer surface from Figure 8, shows a marked decrease of surface hydrophilicity upon increasing the Zn coverage, except for sample TZN_30, where *CA*(*t* = 0) = 21°. This evolution is related to the pronounced hydrophobic character of ZnO nano and microtextured surface (for instance, nanorods), in absence of UV irradiation, following the Cassie-Baxter conditions [50,51]. The TZN_30 exception itself is related to the peculiar rugosity of this sample, in comparison with the other surfaces, as sustained by its morphology data.

To investigate the photo-induced hydrophilic conversion of the ZnO-decorated TiO_2_ nanotube layers, the advancing contact angle (CA) of water with the solid surface was monitored as a function of irradiation UV dose, during the surface activation (Figure 8) for exposure times from 0 to 60 min, while keeping constant light fluence. To monitor the surface wetting condition, which changes with different rates versus the incident UV dose, the advancing CA were measured, following each irradiation instance: (a) every 1 min during the first 4 min interval, (b) every 2 min from 4 to 12 min, (c) every 4 min from 12 to 28 min, and (d) every 8 min from 28 to 60 min.

The hydrophilic conversion of the surface of all the samples tends to saturate after 30 min of irradiation, with CA values below 20°. Therefore, the decrease of surface wettability upon increasing the ZnO coverage should be correlated, in our opinion, with the effect of: (a) a high-rate hydrophilic conversion of the ZnO surface under high-intensity incident UV light exposure, as reported in [51]; (b) a gradual decrease of TiO_2_ UV-exposed area (with net initial super-hydrophilic character [31,52]) at the expense of increasing ZnO coverage. This latter claim is supported by our AFM results: all the samples have similar surface roughness, except for the TZN_30, with surface roughness larger by a factor of two.

It is known that on the bare TiO_2_ nanotube surface, complete spreading of water on the surface is observed, even in low UV irradiation conditions (CA approaching 0°), in contrast to the flat film surface, where initial CA values are around 49°. This agrees with the classical Wenzel wetting model [49], taking into account that almost complete spreading of water takes place on the nanotube surface and in the nanopores. The water intake into the nanopores by capillary forces is favored by the UV exposure, as demonstrated by Kim et al. [53]. This picture changes drastically upon loading the TiO_2_ surface with material of different chemistry and by adding the effects of modified surface texture.

The combined effects of material-related contribution and of larger roughness result in the lowest saturation value of the CA of 13.5° for the sample TZN_30. One can hardly notice any hydrophilic conversion of the bare TiO_2_ surface of nanotube arrays, as well as a decrease of 42% of the CA (sample TZN_90) and 53% (sample TZN_60), when comparing the initial values with their full hydrophylic conversion counterparts.

As mentioned previously, the main goal of our experiments was to evaluate the possibility to extend the durability of the effects of surface photo-activation of TiO_2_ nanotube layers. By synthesizing ZnO/TiO_2_ heterojunctions, these potential achievements can be directly identified and evaluated from the back-reaction experiments, started after complete hydrophilic conversion of the surfaces. Before the back-reaction, the samples were irradiated for 12 h to reach the hydrophilic conversion.

**Figure 9:**
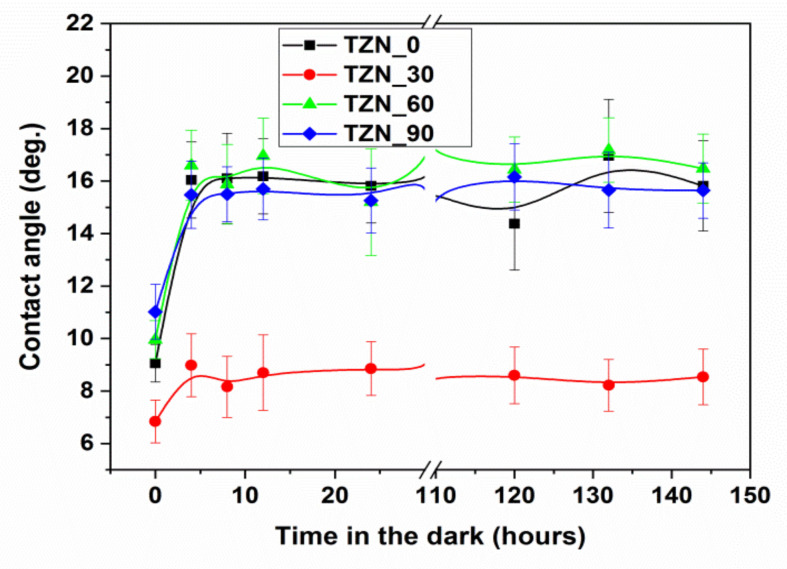
Time evolution of the static contact angle values of the samples during back-reaction.

The time evolution of the static CA during this process was monitored for 144 h, while keeping the layers in dark conditions, and the results are shown in Figure 9. During the first 24 h, the measurements were taken with a step of 4 h and the next 140 h with a step of 12 h.

As one can see from Figure 9, all the samples feature, before the back reaction, super-hydrophilic characteristics (*CA* ≤ 12°). The lowest saturation value of the *CA* (8.5°) after back-reaction is demonstrated by the same sample TZN_30, while the saturation values of all other the samples are situated around 16°. Noteworthy, after surface state recovery during the back-reaction, even after 144 h, the final *CA* values of the high ZnO coverage samples remain lower than the initial *CA* values without UV exposure (see Figure 8) (by a factor of 2.7 for the sample TZN_60 and of 2.1 for TZN_90), indicating that the effects of coupling of the two materials may last much longer than 144 h.

## 4. Conclusions

A two-step procedure was used to synthesize ZnO/TiO_2_ nanotube heterostructures for potential applications in photocatalysis, controlled wetting conversion materials and sensors. In the first step, TiO_2_ nanotubes were prepared by electrochemical anodization of a Ti foil, followed by crystallization annealing of the prepared anodized nanotubes.

The XPS chemical mapping and the data derived from image processing indicate the presence of Ti, O and Zn as the main elements on the material surfaces, with Zn color predominant for the samples with highest ZnO coverage. As revealed by the high-resolution SEM measurements, the TiO_2_ layers contained self-organized uniform tubular arrays, 1.6 μm long, with an average diameter of 88 nm. The as-prepared amorphous layers were subjected to thermal annealing at 450 °C, resulting in a mixture of anatase and small amounts of rutile, with nanocrystal size fluctuating around 25 nm. In the second step, the TiO_2_ nanotube layers were gradually loaded with ZnO nanoparticles originating from an RF magnetron discharge with ceramic ZnO target. This configuration ensured a high-rate, uniform loading of the nanotube layer with ZnO material of several thickness values.

The XPS elemental analysis revealed that the bare TiO_2_ layers contain only TiO_2_ material, covered with contaminant carbon and hydroxil-termination layers. The sputter-cleaning of the surface with Ar^+^ ions evidenced the occurrence of oxygen vacancies due to the ion bombardment and presences of Ti^4+^ to Ti^3+^ and Ti^3+^ states. The as-measured concentration of the reduced species decreased in our experiments upon gradual loading of the nanotubes, mostly due to the screening effect of the loaded material, which obscures the TiO_2_ contribution to the detected XPS signal, due to reduced probing depth of this technique.

The surface photoactivation shows a saturation tendency after 35 min of UV irradiation, with CA values below 20°, with a special remark for the layers with rougher surface. After 144 h of back-reaction, the static CA values (16° for the smoother surface and 8.5° for the rougher surface layer) remain significantly lower than the non-irradiated state. This demonstrates, in our opinion, the synergic effects of material-related morphology and presence of the p-n heterojunctions in extending the photoactivation duration of the ZnO/TiO_2_ nanotube layers.

The current results may serve in designing flexible pathways for fabrication of high-quality devices in the technology of solar cells with TiO_2_ nanotube arrays photoanodes.

## Figures and Tables

**Table 1:** The weight percentage of the anatase phase, the grain size and the root mean square roughness values of the investigated samples.

Sample	RMS Roughness (nm)	D (nm)	W_A_
TZN_0	37.4	26.1	0.702
TZN_30	63.2	25.0	0.727
TZN_60	37.2	22.5	0.706
TZN_90	34.4	22.6	0.726

**Table 2:** Surface atomic concentrations of the main elements in the sputtered samples.

Sample	Ti (at%)	O in O-Ti bonds (at%)	Zn (at%)
**TZN_0**	41.5	58.5	-
**TZN_30**	32.4	58.3	9.3
**TZN_60**	29.6	54.8	15.6
**TZN_90**	25.1	50.7	24.2

**Table 3:** Peak area ratios of O-Ti and O-H bonding and the atomic ratios of Ti^2+^ and Ti^3+^ species with respect to Ti^4+^ in the samples.

Sample	O - Ti/OH	Ti^2+^/Ti^4+^	Ti^3+^/Ti^4+^
**TZN_0**	2.718	0.170	0.400
**TZN_30**	3.805	0.153	0.392
**TZN_60**	4.420	0.120	0.361
**TZN_90**	4.109	0.039	0.261
